# Costal cartilage fractures in blunt polytrauma patients — a prospective clinical and radiological follow-up study

**DOI:** 10.1007/s10140-022-02066-w

**Published:** 2022-06-04

**Authors:** Mari T. Nummela, Tuomo T. Pyhältö, Frank V. Bensch, Mikko T. Heinänen, Seppo K. Koskinen

**Affiliations:** 1 Department of Radiology, Töölö Hospital, HUS Diagnostic Center, Helsinki University Hospital and University of Helsinki, PL 266, 00029 HUS Helsinki, Finland; 2 Department of Orthopaedics and Traumatology, Töölö Hospital, Helsinki University Hospital and University of Helsinki, Helsinki, Finland; 3 Department of Clinical Science, Intervention, and Technology, Division for Radiology, Karolinska Institute, Stockholm, Sweden; 4 Center for Medical Image Science and Visualization (CMIV), Linköping University, Linköping, Sweden

**Keywords:** Costal cartilage fracture, Costochondral fracture, Blunt chest trauma, Post-traumatic calcification

## Abstract

**Purpose:**

To assess the healing of costal cartilage fractures (CCFX) in patients with blunt polytrauma with follow-up imaging and clinical examination. Effect on physical performance and quality of life (QoL) was also evaluated.

**Methods:**

The study group comprised twenty-one patients with diagnosed CCFX in trauma CT. All the patients underwent MRI, ultrasound, ultra-low-dose CT examinations, and clinical status control. The patients completed QoL questionnaires. Two radiologists evaluated the images regarding fracture union, dislocation, calcifications, and persistent edema at fracture site. An attending trauma surgeon clinically examined the patients, with emphasis on focal tenderness and ribcage mobility. Trauma registry data were accessed to evaluate injury severity and outcome.

**Results:**

The patients were imaged at an average of 34.1 months (median 36, range 15.8–57.7) after the initial trauma. In 15 patients (71.4%), CCFX were considered stable on imaging. Cartilage calcifications were seen on healed fracture sites in all the patients. The fracture dislocation had increased in 5 patients (23.8%), and 1 patient (4.8%) showed signs of a non-stable union. Four patients (19.0%) reported persistent symptoms from CCFX.

**Conclusion:**

Non-union in CCFX is uncommon but may lead to decreased stability and discomfort. Both clinical and radiological examinations play an important part in the post-traumatic evaluation of CCFX. CT and MRI visualize the healing process, while dynamic ultrasound may reveal instability. No significant difference in QoL was detected between patients with radiologically healed and non-healed CCFX. Post-traumatic disability was mostly due to other non-thoracic injuries.

**Supplementary Information:**

The online version contains supplementary material available at 10.1007/s10140-022-02066-w.

## Introduction

Blunt chest trauma is common and a major cause of morbidity in hospitalized patients [1–3]. The most common mechanisms of blunt thoracic injury are motor vehicle collisions, motorcycle accidents, and falls. Although most patients with blunt chest trauma suffer from rib fractures (35–40%) [4, 5], costal cartilage fractures (CCFX) are also frequently observed in high-energy trauma CT studies [6]. Fractured cartilage contributes to ribcage instability and may present as prolonged post-traumatic pain and discomfort even months after the initial trauma. Sonography may reveal a CCFX, but cross-sectional imaging (CT, MRI) is essential to verify the clinical suspicion of CCFX. Cartilage fractures are not visible on plain radiographs [7–10]. The healing process of CCFX is not well known; an animal study and a biomechanical study suggest that the union may be unstable [11, 12]. Some case reports revealed prolonged disability due to cartilage injury in contact-sport athletes [8, 13].

For this prospective study, the patients with diagnosed CCFX were summoned for clinical and radiological examinations to evaluate the timeline and natural course of healing. Post-traumatic clinical manifestations and radiological findings of 21 patients with CCFX were evaluated with multimodality imaging (ultra-low-dose CT, MRI, and US), clinical observation and inspection, and two quality of life (QoL) questionnaires. Trauma Registry data were explored to determine clinical variables for these patients. The main aim of this study was to evaluate long-term effects and manifestations of CCFX, to describe typical radiological findings, and to assess impact on QoL.

## Background

Rib fractures are painful and may have an impact on respiratory function. During recuperation from chest trauma, the patients may experience difficulty in performing daily activities and loss of work ability [14]. Shallow breathing due to pain can lead to serious complications. To prevent pulmonary complications, such as respiratory failure, pneumonia, and empyema, aggressive pain management and respiratory support are recommended [15, 16]. These complications are more frequent in patients with multiple rib fractures, and particularly in those with flail chest [17]. The role of CCFX in ribcage instability, post-traumatic pain, and flail chest formation has remained unclear due to sparse data [6, 7, 9]. The general knowledge of CCFX has grown due to increased use of CT and MRI. Plain chest radiographs do not reveal CCFX, as cartilage tissue is radiolucent.

**Fig. 1:**
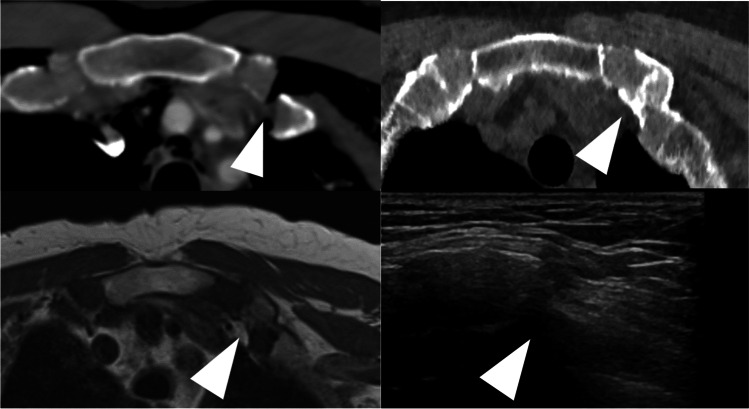
A 64-year-old male with a dislocated costal cartilage fracture on the left 1st rib (arrowhead) from a chest compression injury (left upper axial image). Also, a non-dislocated fracture on the right 1^st^-rib cartilage. Clinical examination revealed no tenderness on palpation around the fracture site, and the patient was asymptomatic. Ultra-low-dose CT (right upper axial image) reveals calcifications (arrowhead) on the fracture site and suggests a stable fracture union. T1-axial MR image (lower left image) shows the cartilage union (arrowhead). Note that there is no fatty infiltration in pectoral muscles. Ultrasound image of the same fracture site (lower right image) shows the step off

Many case reports in athletes have demonstrated detection of costal cartilage injuries with MRI [8, 10, 13, 18–20], but cartilage fractures are also clearly visible on CT (Fig. 1**)** [6, 9, 10]. In whole-body CTs for trauma (WBCT), cartilage injuries may remain overlooked, for example due to perceptual errors or satisfaction of search while reporting multiple injuries in polytrauma patients [6]. Although most blunt chest wall trauma is treated non-operatively, awareness of CCFX adds to the understanding of the extent and volume of thoracic injury and ribcage instability [10]. The late consequences of blunt chest trauma include pain, sense of ribcage stiffness, snapping sensation, loss of respiratory volume, and restrictions in physical activity [14]. At our institution, blunt chest trauma patients have clinical follow-up consultations with trauma surgeons 1 and 6 weeks after release from care. In general, follow-up imaging consists of plain radiographs; CT imaging is reserved for further evaluation of clinical complications.

## Materials and methods

### Patients

This study was conducted in a level I trauma center in a metropolitan area of 1.8 million people. Institutional review board approval was granted by the Ethics Committee of the Department of Surgery for clinical follow-up, questionnaires, and imaging studies. In a previous retrospective study, the patients with CCFX were identified from WBCT examinations for trauma from the hospital picture archiving and communication systems, Agfa IMPAX 6.0 (Agfa HealthCare NV, Mortsel, Belgium). All the patients who entered the ER with a history of blunt trauma, were > 18 years of age, and underwent WBCT for trauma during the years 2013–2015 were included. From this study cohort, 114 patients with CCFX were identified. Of these, 8 patients died during the first 30 days after trauma [SI 1] and 5 died between the date of leaving the hospital and the beginning of data collection (15 April 2017). Additionally, 38 patients were not reachable due to location or health issues. The patients with pacemakers (2) were excluded due to restrictions in MRI imaging, as there was no cardiologist available on site to adjust the pacemaker during research imaging hours. In addition, 14 patients with CCFX identified from the year 2016 WBCTs for trauma were also invited to this prospective study to evaluate chronologically more recent fractures [SI 2].

Invitation letters were sent to 75 patients (61 [2013–2015] and 14 [2016]), 21 (28.0%) patients participated in this study (16 of 61, 26.2% and 5 of 14, 28.6%). The patients were invited via regular mail. The letter included a cover letter with a description of patient selection, basic principles of the study, and contact information; study information, and patient consent form approved by the ethics committee; a standardized RAND SF36 QoL questionnaire [21, 22]; a dedicated QoL questionnaire for patients with blunt thoracic trauma (designed by authors) [SI 3]; and an MRI safety questionnaire as an institutional standard procedure. All the patients had the right to discontinue the study and withdraw their consent; all enrolled subjects completed the entire study protocol.

### Clinical control and Trauma Registry data

An orthopedic trauma surgeon with > 14 years of clinical experience evaluated the current physical status, disability, and ranges of movement (ROM). Ribcage symmetry and chest wall movements during the breathing cycle were visually examined. Breathing sounds were bilaterally evaluated. Entire chest wall, clavicles, scapulas, shoulder areas, and the injured parts of the chest wall were manually palpated to detect focal tenderness. The ROM in glenohumeral joints was analyzed and the underlying reason for possible diminished ROM was evaluated.

Trauma Registry data were explored to determine mechanism of injury (MOI), New Injury Severity Score (NISS), and Injury Severity Score (ISS), intensive care unit length of stay (ICU LOS), university hospital length of stay (LOS), need for mechanical ventilation, and duration of ventilation.

### Imaging

The chest with focus on the area of injured cartilage (identified in the initial WBCTs for trauma) was imaged with all three different modalities for each patient as follows: (1) ultrasound (GE Logiq E9, GE Healthcare, Chicago, IL), (2) ultra-low-dose-CT; ULD-CT (Discovery 750HD, 64-detector row CT scanner, GE Healthcare), and (3) 1.5 T MRI (MAGNETOM Aera, 1.5 T, Siemens Healthcare GmbH, Erlangen, Germany). Imaging was performed without intravenous contrast media administration.

Ultrasound (US) images of the injured cartilage were obtained in conjunction with the physical examination. The following types of broad-spectrum linear transducers were used: 9L-D (2–8 MHz, FOV 44 mm) and 12L-RS (5–13 MHz, FOV 39 mm). US was used to identify the costochondral junction and the cartilage fracture site. Doppler US was used to identify increased vascularity at the fracture site. Sonar palpation was used to indicate movement in the fracture line.

A non-contrast ULD-CT (sub-mSv) protocol with iterative reconstruction (VEO, GE Healthcare) was used to ensure minimal radiation in imaging of a benign condition. Three-plane orthogonal reformatted image stacks (axial, sagittal, coronal) were used for evaluation.

MRI images (five sequences) were obtained in prone position (free respiration, non-gated) with a body-coil. Three sequences were imaged in the axial plane from the area of injured cartilage (T2-fat-saturated axial, T2 axial, and T1 axial). T1 sequence was used to evaluate fatty infiltration of the pectoralis muscles. Two sequences were obtained in the coronal plane of the anterior chest wall (STIR coronal or PD fat-saturated coronal and T2 coronal). The orientation was coronal oblique — parallel to the sternum (Appendix, Fig. 5). MRI imaging was performed by an experienced radiology technician and monitored directly by at least one of the radiologists involved in this study.

### Image interpretation

US images were evaluated by the radiologist performing the examination. Both ULD-CT and MRI images were interpreted separately by two radiologists, who were blinded to each other and to physical exam results. Fracture healing was evaluated by assessment for noticeable fracture line, persistent edema on fracture site, presence and degree of calcification, and increased or decreased fracture dislocation. Equivocal findings were settled by consensus readout, which was used in the final analysis.

### Quality of life questionnaires

Two survey instruments were used in this study to evaluate health-related QoL and degree of disability after trauma. All the patients participating in this study received a standardized RAND 36 questionnaire (36-Item Short Form Survey Instrument [SF-36]) [21, 22]. In addition, a dedicated thoracic trauma questionnaire [SI 3] was written to evaluate limitations and symptoms particularly due to thoracic injury. The results from the RAND 36 questionnaire were compared between the patients with healed and non-healed costal cartilage fractures.

### Statistical analyses

The interobserver agreement for imaging findings was determined using Cohen’s kappa (κ). The κ-values were considered as follows: 0.01–0.20 slight agreement, 0.21–0.40 fair agreement, 0.41–0.60 moderate agreement, 0.61–0.80 substantial agreement, and 0.80–0.99 almost perfect agreement. Proportion of agreement (POA) was also used to test agreement of dichotomous evaluation between readers (interobserver variation). The strength of POA was determined as follows: 0.0–0.2 poor, 0.21–0.4 fair, 0.41–0.6 moderate, 0.61–0.8 good, and 0.81–1.0 excellent [23].

Statistical analyses were performed using a commercial software package SAS/STAT v.9.4 (SAS Institute Inc., Cary, NC, USA).

## Results

### Patients

All the participants (21) were male, and age ranged from 30 to 77 (mean 59.8 years, median 63 years). The most common mechanism of injury was a fall from a height (7/21, 33.3%) (Tables 1 and 4). The mean time from injury to follow-up examination was 34.1 months (median 36, range 15.8–57.7). Fourteen (66.7%) had multiple costal cartilage fractures.

**Table 1:** Variables from Trauma Registry data

Variable	Patients included in follow-up	
	*n* (%)	Range/%
*n*	21	
Age (mean)	59.8	30–77
Gender		
Male	21	100%
Female	0	0
ISS	25.5	17–38
NISS	31.9	17–43
NISS < 16 (*n*)	7 (33.3%)	
MOI (*n*)		
Traffic, motor vehicle	3	14.3%
Traffic, motorcycle	1	4.8%
Traffic, bicycle	2	9.5%
Traffic, pedestrian	0	0.0%
Stab	0	0.0%
Struck	2	9.5%
Low fall	1	4.8%
High fall	4	19.0%
Other	1	4.8%
Intubated (*n*)		
No	6	28.6%
Yes, on field	2	9.5%
Yes, in ER	1	4.8%
Yes, post-ER	5	23.8%
Ventilation duration (days)	4.9 (mean)	1–16
ICU LOS (days)	8.6 (mean)	1–17

*ISS* Injury Severity Score, *NISS* New Injury Severity Score, *MOI* mechanism of injury, *ER* emergency room, *ICU LOS* intensive care unit length of stay

### Clinical status

Four patients (19.0%) complained of persisting pain or discomfort at the fracture site. In 3 patients, fractures were evaluated as non-healed on CT, and these patients had focal tenderness on clinical exam. Ribcage asymmetry was found in 2 patients (9.5%). Seven patients (33.3%) reported focal tenderness on clinical examination. Fracture site was palpable in 7 patients (33.3%); 3 presented with focal tenderness. Two patients reported persistent pain from scapular fracture and 1 from multiple rib fractures. Two patients had a diminished ROM, 1 in the entire rib cage, and the other due to scapular fracture. There were no clinical signs of restricted respiration and no signs of involvement of accessory breathing muscles (Table 2).

**Table 2:** Results from clinical examination

Nr	Gender	Age	MOI	NISS	ISS	TP mo#	Tenderness	Clinical status
1	M	66	Fall	17	17	46.1	No	Clicking and discomfort 6 months post-trauma, now asymptomatic
2	M	64	compr	43	38	57.7	No	Stable, no pain
3	M	61	MVA	22	19	45.3	No	Palpable fracture site, no pain
4	M	60	MCA	17	17	42.2	No	Stable, no pain
5	M	55	BCA			53.1	Yes	Palpable fracture site pain and discomfort from scapular fx
6	M	72	Fall	29	29	40.0	No	Stable, no pain
7	M	63	Fall	43	35	46.3	No	Stable, no pain
8	M	30	BCA			33.0	No	Clicking 1 month post-trauma, now discomfort in heavy breathing*
9	M	31	MVA	34	29	37.0	No	Stable, no pain
10	M	47	compr	34	29	36.3	No	Stable, no pain
11	M	66	compr	36	29	36.5	No	Stable, no pain
12	M	61	MVA			36.0	No	Unstable left CC 8–9
13	M	62	BCA	27	19	28.8	Yes	30% limitation in range of motion in thorax
14	M	68	MVA			28.7	No	Stable, no pain*
15	M	77	BCA	27	22	28.8	No	Stable, no pain
16	M	47	Fall			25.1	No	Stable, no pain from rib cage, discomfort from scapular fx
17	M	48	Fall			21.2	Yes	Stable, no pain
18	M	71	Fall	34	20	19.4	Yes	Stable, pain in right CC 4, CC1 healed*
19	M	70	MVA	43	38	19.8	No	Stable, no pain, prominent fracture site*
20	M	67	Fall	41	16	16.8	Yes	Pain in right CC 1–3, right flank sore, palpable pain in multiple rib fxs*
21	M	69	MCA			15.8	No	Stable, no pain, right-sided ribcage impression, multiple rib fx healed

*MOI* mechanism of injury, *NISS* New Injury Severity Score, *ISS* Injury Severity Score, *BCA* bicycle accident, *MVA* motor vehicle accident, *MCA* motorcycle accident, *CC* costal cartilage, *fx* fracture, *compr* compression

^*^Clinical status in bold font was evaluated as non-healed on imaging

^#^Timepoint of clinical status and follow-up imaging with ULD-CT in months. Time calculated from initial trauma CT as baseline

### Imaging

All the patients underwent all three different modalities in follow-up imaging. The imaging intervals are stated in Table 4. Most costal cartilage fractures (15/21, 71.4%) were radiologically evaluated as healed (κ 0.69 (95% CI 0.3771–1.000) (Fig. 3). The results from the image evaluation are shown in Table 3.

**Table 3:** Results from consensus reading of follow-up imaging

Variable	Consensus reading	POA (%)	Kappa (95% CI)
ULD-CT			
Healed	15/21 (71.4%)	85.7	0.69 (0.38–1.00)
Partially healed	1/21 (4.8%)	95.2	0.64 (0.0079–1.00)
Fracture line visible	9/21 (42.9%)	95.2	0.90 (0.72–1.00)
Fracture dislocation or reposition	6/21 (28.6%)	76.2	0.30 (− 0.18–0.77)
Calcification on fracture site	9/21 (52.4%)	66.6	0.34 (− 0.04–0.72)
Newly detected fracture	5/21 (23.8%)	85.7	0.58 (0.15–1.00)
MRI			
Edema visible on MRI	8/21 (38.1%)	61.9	0.11 (− 0.29–0.50)
Fracture line visible on MRI (T2)	20/21 (95.2%)	76.2	− 0.08 (− 0.22–0.05)
Muscle atrophy (MA) on MRI (T1)	0	100	1.00
MA compared to initial trauma CT	0	100	1.00
Newly detected fracture	3/21 (14.3%)	81	0.42 (− 0.0011–0.83)
Ultrasound			
Fracture line visible	13/21 (61.9%)	61.9	0.29 (− 0.04–0.63)
Fracture dislocation	14/21 (66.7%)	71.4	0.33 (− 0.07–0.73)
Calcification	5/21 (23.8%)	76.2	0.49 (0.13–0.86)
Movement in fracture site	1/21 (4.8%)	100	1.00

*POA* proportion of agreement, *ULD-CT* ultra-low-dose CT

Sonography was most useful in dynamic evaluation and detected cartilage movement and dislocated fractures (Appendix, Fig. 3). Instability was perceived on dynamic US and during clinical examination in 1 patient (4.7%). Movement was detected in dynamic US examination while compressing the fracture area with the US probe. First-rib costochondral synostosis was not an optimal site for US examination due to its location deep in the clavicula and sternoclavicular joint and since the first rib cartilages are usually heavily calcified on the surface. No extra benefit from the use of Doppler US was noted, and no vascular changes in the damaged areas were detected.

Initially, fractures were detected in 10 patients (47.6%). Ultra-low-dose-CT examination showed calcifications around the initial fracture site in all the patients (Fig. 1). These post-traumatic calcifications differ from physiological cartilage calcifications in morphology and orientation (Appendix, Fig. 4). Post-traumatic calcifications are found vertically oriented in the fracture line and horizontally following the perichondrium. The physiological calcifications are patchy, more randomly distributed, and parallel to the long axis of the rib.

In comparison with the initial trauma CT study, unsuspected increased dislocation of the evaluated or adjacent CCFX was found in 5 patients (23.8%). Initially, dislocated fractures were found in 10 patients (47.6%) (Table 4). ULD CT imaging of the thorax had a mean dose length product (DLP) of 32 (range 15–58) when compared to the initial trauma WBCT DLP of 1668 (range 516–4486) (Table 4). Ultra-low-dose imaging provided sufficient image quality for evaluating the post-traumatic changes of the bony ribcage (Fig. 1). Fracture lines remained visible on CT in 6 patients (28.6%).

**Table 4:** TE DLP values from initial trauma WBCT and ULD CT. Imaging intervals between the modalities and initial trauma CT. Fracture site, fracture dislocation, detection rate, and status on healing

nr	Age	NISS	ISS	MOI	TE DLP (WBCT)#	TE DLP (ULD CT)#	TP ULD CT	TP MRI	TP US	fx site	disloc	Detection	Healed
1	66	17	17	Fall	1693	47	46.13	45.97	46.0	R6	No	Yes	Yes
2	64	43	38	compr	4211	28	57.70	56.93	56.9	L1-2	Yes	No	Yes
3	61	22	19	MVA	2862	29	45.33	45.03	45.3	R3-7, L6-7	Yes	No	Yes
4	60	17	17	MCA	4486	58	42.23	41.87	42.1	R6	No	Yes	Yes
5	55			BCA	1908	33	53.13*	40.50*	53.1*	L3	No	No	Yes
6	72	29	29	Fall	668	16	40.00	39.67	39.9	L6-7	Yes	No	Yes
7	63	43	35	Fall	1283	28	46.30	46.73	46.3	R3	No	No	Yes
8	30			BCA	1245	22	33.00	32.83	32.9	L1	Yes	Yes	No
9	31	34	29	MVA	2330	30	36.97	37.07	37.0	R5-7	No	Yes	Yes
10	47	34	29	compr	2526	37	36.27	36.33	36.3	R7	No	No	Yes
11	66	36	29	compr	516	25	36.47	36.43	36.5	L5-7	Yes	Yes	Yes
12	61			MVA	1113	27	36.00	35.97	36.0	L8	No	No	Yes
13	62	27	19	BCA	844	28	28.80	28.90	28.8	R2-4	No	Yes	Yes
14	68			MVA	1449	56	28.73	28.80	28.7	R1	No	Yes	No
15	77	27	22	BCA	1481	42	28.83	28.80	28.8	R6-7	No	No	Yes
16	47			Fall	1037	23	25.13	25.07	25.1	R1-2, L1	Yes	Yes	Yes
17	48			Fall	1886	56	21.23	21.40	21.4	R8-9	No	No	Yes
18	71	34	20	Fall	320 (THX)	21	19.37	19.97	19.4	R1	Yes	No	No
19	70	43	38	MVA	692	16	19.80	19.03	19.8	R7	Yes	No	No
20	67	41	16	Fall	1009	18	16.83	17.00	16.8	R1-4, L1-2	Yes	Yes	No
21	69			MCA	1473		15.83	15.97	16.0	R1	Yes	Yes	Yes

*MOI* mechanism of injury, *NISS* New Injury Severity Score, *ISS* Injury Severity Score, *TE DLP* total estimated dose length product, *TP* time point between follow-up imaging and initial CT in months, *Fx* fracture, *disloc* dislocated fracture, *BCA* bicycle accident, *MVA* motor vehicle accident, *MCA* motorcycle accident, *compr* compression

^*^in one patient imaging was delayed due to other health issues

^#^TE DLP unit: mGy × cm

**Fig. 2:**
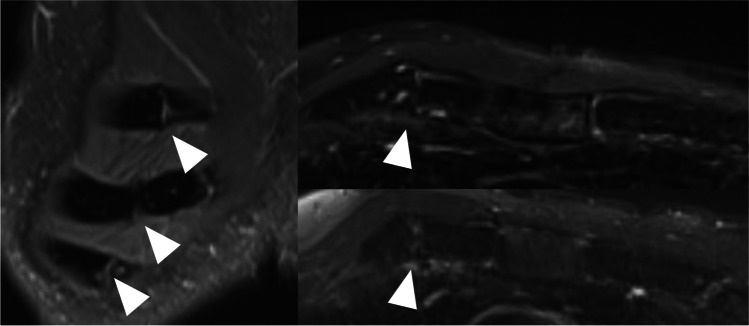
A 61-year-old male from a fall. Non-healing costal cartilage fractures (arrow head) on coronal TIRM image, ribs 2–4 (left image). Axial T2-fat saturated images of costal cartilage in ribs 2 and 3 (images on the right)

**Fig. 3:**
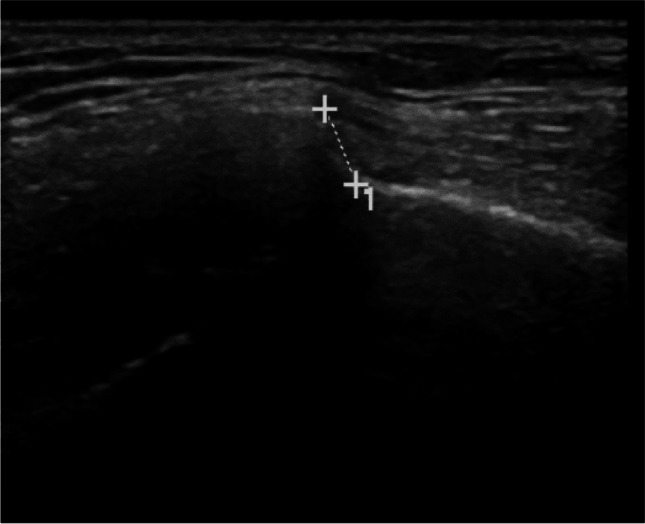
Step off indicating dislocated fracture on the cartilage surface as seen on ultrasound

**Fig. 4:**
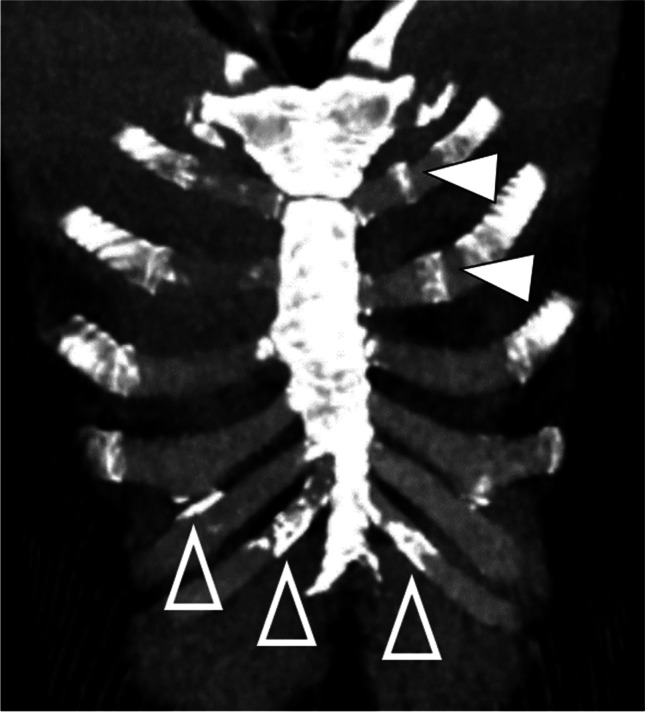
A 55-year-old male. Healed midchondral fractures of the left 2nd and 3rd ribs show calcifications on follow-up CT (coronal plane). Post-traumatic calcifications (arrowhead) and physiological calcifications (open arrowhead)

**Fig. 5:**
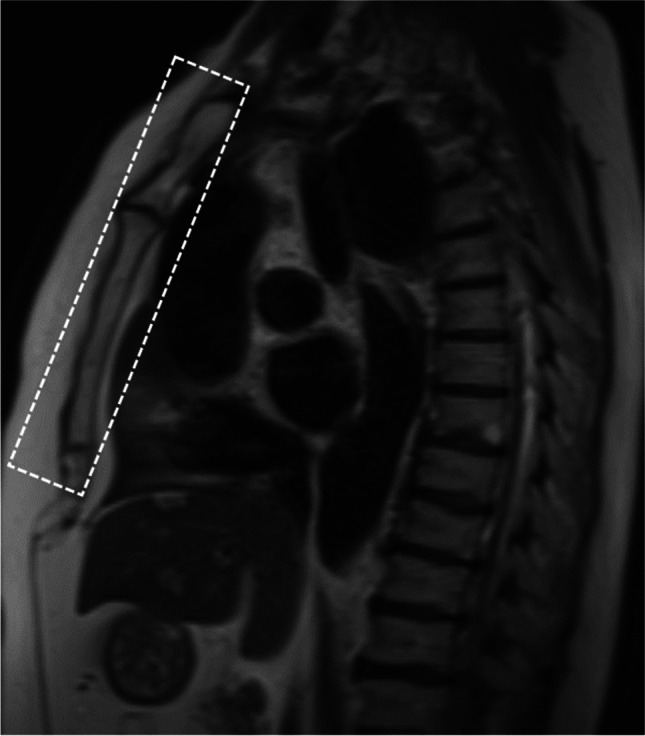
Orientation of MRI images was coronal oblique

MRI image acquisition was performed with a body-coil during free breathing. The patients were lying face down to reduce anterior chest wall movement artifacts. Only one patient reported discomfort at the end of the series of images taken in prone position. All the adequate images had been taken at that point. T2-weighted images were considered most useful in evaluating cartilage and their continuity (Fig. 2). Fat-saturated T2-weighted images showed persistent edema at the fracture site in 8 patients (38.1%) (Fig. 2). No atrophy or fatty degeneration of the pectoralis muscles was detected on T1-weighted images (Fig. 1).

### Quality of life questionnaires

No statistically significant difference of RAND36 scores between radiologically healed and non-healed patients was detected. In the dedicated chest trauma survey, 9 patients (42.9%) reported no thoracic pain. In 7 patients (33.3%), thoracic pain affected sleep, while in 13 (61.9%) pain from other body regions affected sleep.

Most patients had chest-related symptoms. The most common symptoms were chronic cough (14 patients, 66.7%), difficulties with deep inhalation (12 patients, 57.1%), difficulties with strenuous exercise (12 patients, 57.1%), local pain (7 patients, 33.3%), tingling or numbness (5 patients, 23.8%), and a snapping sensation (5 patients, 23.8%). Ten (47.6%) patients used pain medication daily, most commonly paracetamol (5 patients, 23.8%) and NSAIDs (4 patients, 19.0%). Four (19.0%) patients reported no pain at all. Other symptoms affecting daily life were pain or dysfunction of the lower extremities in 7 patients (33.3%) and spine in 9 patients (42.9%).

### Trauma Registry data

Seven patients (33.3%) were not included in the Trauma Registry as their ISS score ≤ 16. For the remaining 14 patients (66.7%), mean NISS score was 31.9 (median 34, range 17–43) and mean ISS score was 25.5 (median 25.5, range 17–38). The mean ICU length of stay (ICU-LOS) was 8.6 days (median 8.5, range 1–17). Mean university hospital LOS was 14.6 days (median 13, range 7–30). Eight of these 14 patients (57.1%) needed mechanical ventilation for mean duration of 8.5 days (range 1–16) (Table 1).

## Discussion

Detection of costal cartilage fractures has increased due to increasing use of cross-sectional imaging by CT and MRI. As a result of the healing process, calcifications commonly develop at the fracture site and are visible on CT. Fracture dislocation can increase over time and persistent movement can be detected on dynamic US. MRI is best suited for visualizing local persistent edema. Clinical examination reveals focal tenderness and a palpable fracture site. In general, most CCFX will heal over time and become asymptomatic. However, some patients report pain, discomfort, and a snapping sensation around the costal cartilage fracture even years after the initial trauma. A diagnostic work-up that consists of both clinical examination and imaging with CT or MRI ensures a comprehensive evaluation. Most, but not all, fractures that were radiologically evaluated as non-healed were symptomatic. Conversely, some symptomatic fractures were evaluated as healed on imaging. Thus, the combination of clinical examination and imaging will help assess the overall situation.

As costal cartilage injuries are commonly seen in high-energy trauma, other concomitant injuries add to the pain and disability load, and cartilage injuries may remain overlooked as the source of pain and discomfort.

This study had some limitations. Traumatic changes were not limited to the thorax; most patients had additional severe injuries. Only patients without significant physical disability accepted the invitation and were able to attend the appointments without any assistance. There is a possibility that patients who were more severely affected or restricted by their injuries might have chosen to not participate in this follow-up study, implicating selection bias. Another bias was created from the motivation of the patients to participate. Most of the patients who decided to participate in this study had prolonged symptoms from the original trauma; some had symptoms outside the thoracic region. As the main aim of this study was to examine the healing process of CC fractures and describe the clinical and radiological post-traumatic changes, any patient that was previously diagnosed with a costal cartilage injury was considered valuable to this study. The number of potential participants for the study was limited mostly by injury incidence and patient’s willingness to participate.

Follow-up imaging was performed on average 3 years after the initial trauma, which we consider sufficient for the evaluation of long-term effects. This interval was an estimate based on the general experience of trauma radiologists and trauma surgeons included in this study with reference to how osseous rib fractures tend to heal. A more dedicated follow-up protocol with preset intervals for follow-up imaging (e.g., 3, 6, 12, and 24 months) may provide more detailed information about the healing process. We recommend that in patients with persistent symptoms (pain, discomfort) in the anterior chest wall, MRI, and CT follow-up imaging would give information about the current status of the fractures. Clinical examination made at the same time would provide additional information. In selected cases with severely dislocated/unstable fractures, operative treatment might be considered.

For this study, we chose ULD-CT, MRI, and US for imaging because of their respective strengths and to compensate for weaknesses. Different imaging modalities provide complementary advantages regarding cartilage injury diagnosis and follow-up. ULD-CT has a very low radiation dose but is well suited for evaluating degree of dislocation and presence of cartilage calcifications. Overall, CT visualizes osseous and cartilaginous structures and fracture lines very well, whereas fracture instability can be visualized on dynamic US. MRI is best suited for evaluating soft-tissue changes such as edema and muscle atrophy. Movement artifacts may affect image quality on MRI, which is dependent largely on patient co-operation, scanners, and imaging protocols.

POA in addition to κ were used to express interobserver agreement, as κ is affected by the prevalence of the finding under observation. For rare findings, such as in “no fracture line visible on MRI T2” in this study, very low κ values may not necessarily reflect low rates of overall agreement [24, 25]. The interobserver agreement and proportion of agreement were good on ULD-CT studies.

Visible fracture line can be detected on US, CT, or MRI. MRI shows persistent edema on the fracture site and possible dislocation. T1-weighted images illustrate fatty degeneration or atrophy of muscles. The primary value of US was in detecting fracture instability on dynamic examination. US is inherently observer-dependent, which necessarily affects reproducibility. Standardized imaging planes on CT and MRI avoid this limitation by providing easily comparable imaging data.

## Conclusions

Non-union in CCFX is uncommon but may lead to decreased stability and discomfort. Both clinical and radiological examinations play an important part in the post-traumatic evaluation of CCFX. CT and MRI visualize the healing process, while dynamic ultrasound may reveal instability. No significant difference in QoL was detected between the patients with radiologically healed and non-healed CCFX. Post-traumatic disability was mostly due to other non-thoracic injuries.

### Electronic supplementary material

Below is the link to the electronic supplementary material.

Supplementary file1 (PDF 221 KB) [SI 1] 30-day mortality in primary group

Supplementary file2 (PDF 553 KB) [SI 2] Flowchart of included and excluded patients

Supplementary file3 (PDF 901 KB) [SI 3] Dedicated Chest Trauma Questionnaire

## Appendix

Figure 3

Figure 4

Figure 5
