# Supplementary material for: Costal cartilage fractures in blunt polytrauma patients — a prospective clinical and radiological follow-up study
Source: Emerg Radiol. 2022 Jun 4;29(5):845–54. doi: 10.1007/s10140-022-02066-w (PMC9458556; doi:10.1007/s10140-022-02066-w)
Supplement: Supplementary file 1 — Supplementary file1 (PDF 221 KB) [SI 1] 30-day mortality in primary group [file 10140_2022_2066_MOESM1_ESM.pdf]

30-day mortality in primary study group.

	age	gender	NISS	ISS	MOI	GCS prim	BP prim	BE prim	LOS	LOS ICU	Intub.	Vent. days	cause of death
1	59	M	22	17	BCA	n/a	149	-0.5	2	2	yes	2	brain injury
2	85	M	22	22	MVA	n/a	105	-5.9	5	0	yes	0	brain injury
3	48	M	27	27	MVA	3	0	-26.6	0	0	yes	0	massive bleed
4	38	M	48	41	MCA	n/a	85	-8.5	5	5	yes	5	brain injury
5	31	M	50	50	MCA	3	80	-9.6	2	2	yes	2	brain injury
6	62	M	59	54	BCA	3	132	-2.6	6	6	yes	6	brain injury
7	41	M	66	59	Fall	8	96	-13.3	9	9	yes	9	pulmonary embolism
8	50	F	66	66	Fall	n/a	74	n/a	0	0	yes	1	brain injury

30-day mortality in primary study group.

NISS = New Injury Severity Score. ISS = Injury Severity Score. MOI = mechanism of injury. BCA = bicycle accident. MVA = motor vehicle accident. MCA = motorcycle accident. GCS = Glasgow Coma Scale. BP = systolic blood pressure. BE = base excess. LOS = length of stay. LOS ICU = intensive care unit length of stay
