# Supplementary figures and images for: Costal cartilage fractures in blunt polytrauma patients — a prospective clinical and radiological follow-up study
Source: Emerg Radiol. 2022 Jun 4;29(5):845–54. doi: 10.1007/s10140-022-02066-w (PMC9458556; doi:10.1007/s10140-022-02066-w)

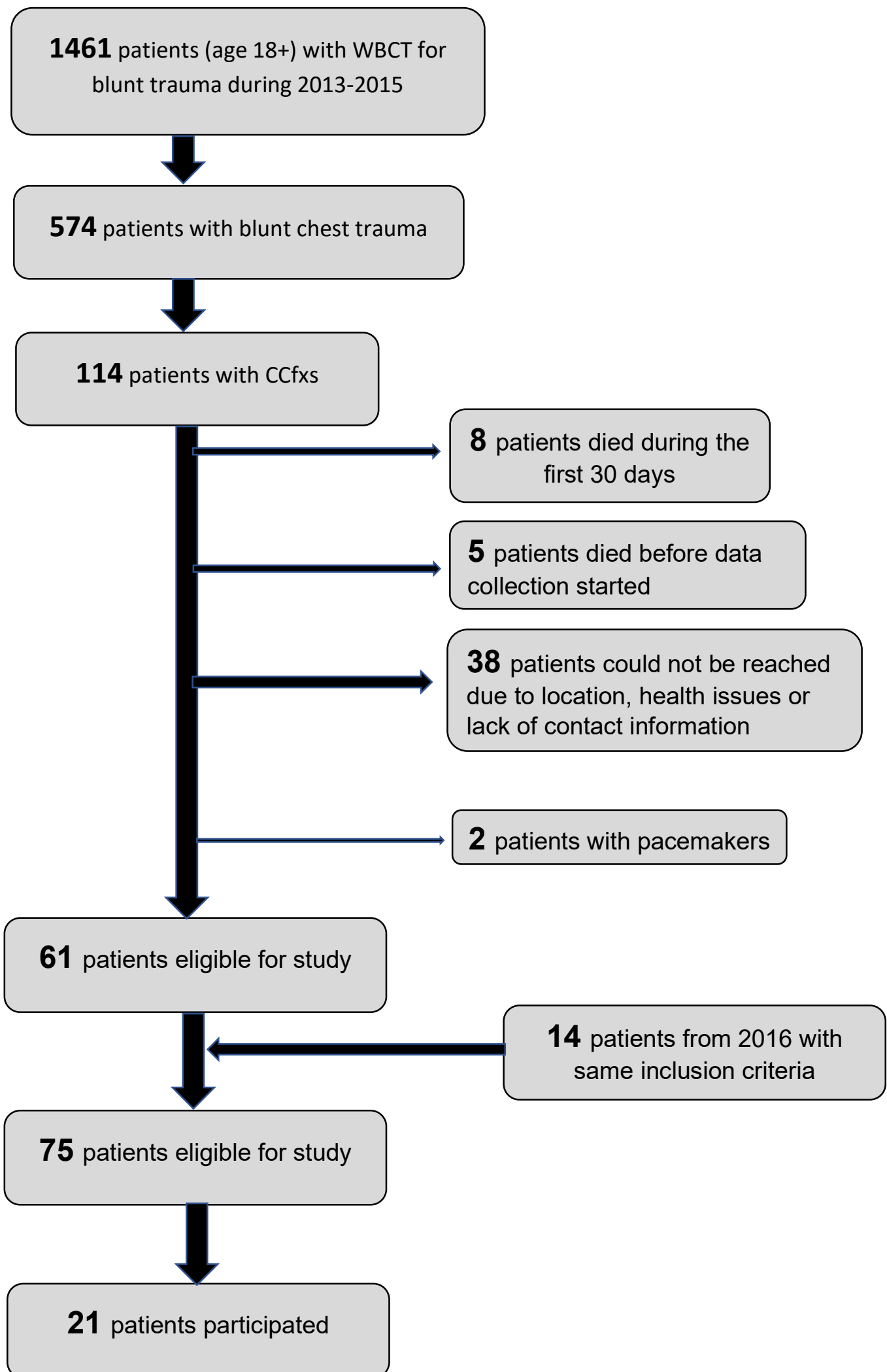

Supplement: Supplementary file 2 — Supplementary file2 (PDF 553 KB) [SI 2] Flowchart of included and excluded patients [file 10140_2022_2066_MOESM2_ESM.pdf]
