# Supplementary material for: Costal cartilage fractures in blunt polytrauma patients — a prospective clinical and radiological follow-up study
Source: Emerg Radiol. 2022 Jun 4;29(5):845–54. doi: 10.1007/s10140-022-02066-w (PMC9458556; doi:10.1007/s10140-022-02066-w)
Supplement: Supplementary file 3 — Supplementary file3 (PDF 901 KB) [SI 3] Dedicated Chest Trauma Questionnaire [file 10140_2022_2066_MOESM3_ESM.pdf]

**Chest Trauma Instrument: Questionnaire for study "Clinical and radiological follow up study on costal cartilages in blunt polytrauma patients" (version 31st March 2016)**

Questions regarding three areas of interest – 1) pain associated with chest trauma, 2) functional status, 3) quality of life. All given information is strictly confidential.

| | |
|---|---|
| Date | |
| Name | |
| ID nr | |
| Address | |
| Phone | |
| Date of trauma | |
| Investigator | |

Do you use medication to control the pain? (circle the appropriate response) YES NO

If "YES", what kind of medication (name, dosage and administration route: oral, injection, patch) and how often (how many times per day/week/month etc)?

.......................................................................................................................................

.......................................................................................................................................

Where is the pain located? (mark the area/s in the drawing)

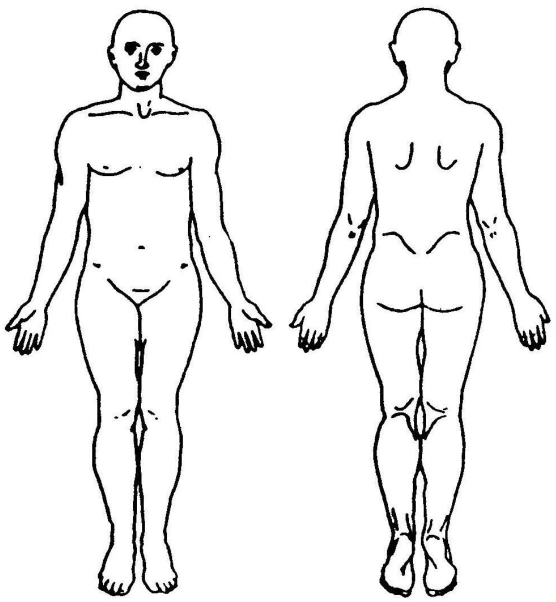

**Please circle the appropriate number** (often = daily)
If there is no appropriate response, you can leave the number uncircled

1. Do you suffer from pain in the chest area? never 0 1 2 3 4 5 often

2. Do you have trouble sleeping due to pain on the chest? never 0 1 2 3 4 5 often

3. Do you have trouble sleeping due to other pain? never 0 1 2 3 4 5 often

4. Do you suffer from coughing? never 0 1 2 3 4 5 often

5. Have you felt a snapping sensation on the chest wall? never 0 1 2 3 4 5 often

6. Do you have discomfort in breathing deeply? never 0 1 2 3 4 5 often

7. Do you have trouble in dressing/undressing yourself? never 0 1 2 3 4 5 often

8. Have you felt a sensation of weight or discomfort on the chest during exercise?

   never 0 1 2 3 4 5 often

9. Have you felt a sensation of weight or discomfort on the chest during rest?

   never 0 1 2 3 4 5 often

10. Have you had difficulties in sports/exercise that gets you out of breath?

    never 0 1 2 3 4 5 often

11. Have you felt a sensation of numbness, tingling or stinging in the chest?

    never 0 1 2 3 4 5 often

| | | | |
|---|---|---|---|
| 12. Do you smoke | Daily ☐ | Occasionally ☐ | Never ☐ |

If you do smoke, how many cigarettes per day? ____________

13. My current health is good agree 0 1 2 3 4 5 do not agree
